# Deep Transfer Learning for Time Series Data Based on Sensor Modality Classification

**DOI:** 10.3390/s20154271

**Published:** 2020-07-31

**Authors:** Frédéric Li, Kimiaki Shirahama, Muhammad Adeel Nisar, Xinyu Huang, Marcin Grzegorzek

**Affiliations:** 1 Institute of Medical Informatics, University of Lübeck, Ratzeburger Allee 160, 23538 Lübeck, Germany; muhammad.nisar@student.uni-luebeck.de (M.A.N.); huang@imi.uni-luebeck.de (X.H.); grzegorzek@imi.uni-luebeck.de (M.G.); 2 Department of Informatics, Kindai University, 3-4-1 Kowakae, Higashiosaka City, Osaka 577-8502, Japan; shirahama@info.kindai.ac.jp

**Keywords:** transfer learning, deep learning, time-series classification, wearable computing, human activity recognition, emotion recognition

## Abstract

The scarcity of labelled time-series data can hinder a proper training of deep learning models. This is especially relevant for the growing field of ubiquitous computing, where data coming from wearable devices have to be analysed using pattern recognition techniques to provide meaningful applications. To address this problem, we propose a transfer learning method based on attributing sensor modality labels to a large amount of time-series data collected from various application fields. Using these data, our method firstly trains a Deep Neural Network (DNN) that can learn general characteristics of time-series data, then transfers it to another DNN designed to solve a specific target problem. In addition, we propose a general architecture that can adapt the transferred DNN regardless of the sensors used in the target field making our approach in particular suitable for multichannel data. We test our method for two ubiquitous computing problems—Human Activity Recognition (HAR) and Emotion Recognition (ER)—and compare it a baseline training the DNN without using transfer learning. For HAR, we also introduce a new dataset, Cognitive Village-MSBand (CogAge), which contains data for 61 atomic activities acquired from three wearable devices (smartphone, smartwatch, and smartglasses). Our results show that our transfer learning approach outperforms the baseline for both HAR and ER.

## 1. Introduction

The prevalence of wearable devices has simplified the collection of sensor data for ubiquitous and wearable computing applications over the past years. In such context, machine learning has become necessary to provide meaningful services by automatically recognising complex patterns in time-series data. Following the most common approach, an ubiquitous computing application—like Human Activity Recognition (HAR) or Emotion Recognition (ER)—is formulated into a classification problem. A classification model is built on a training dataset composed of sensor data labelled with their corresponding classes (e.g., activities and emotions for HAR and ER, respectively). The model is then used to estimate the class of test data whose actual class is unknown.

To build an accurate model, it is required to find an appropriately abstracted representation of data—called features—which would contain all the information relevant to the target classification problem. This process is referred to as feature extraction. In traditional approaches, features were heuristically engineered based on prior knowledge about sensor data in the target problem. They have however been progressively overshadowed by feature learning methods which learn useful features on data in a more automated way [1,2]. The most popular feature learning methods are based on deep learning, i.e., machine learning using Deep Neural Networks (DNNs). A DNN consists of an ensemble of artificial neurons organised in a layer-wise fashion. Each neuron is a simple nonlinear computational unit with internal parameters, weights, and biases. During the training of a DNN, these parameters are optimised so that the model can accurately categorise training data into their own classes. Past works have shown that neurons of a trained DNN encode specific features which are more effective than traditional human-crafted features [3]. The effectiveness of DNNs has been consistently verified over the past years for numerous wearable-computing applications, including HAR [1,2,4,5] and ER [6,7].

Using DNNs is however confronted to several difficulties in practice, such as lack of practical technique for the optimisation of hyper-parameters (e.g., neural activation function, number of layers, number of neurons per layer, etc.), requirements in high computational power to train complex models in a reasonable amount of time, etc. Among them, the major obstacle remains the need for a large quantity of labelled training data. A high diversity in the training data are required so that the classification model becomes robust to the intra-class variability which might be caused by many different factors. For HAR for instance, a way to execute a certain activity may significantly vary depending on persons, producing very different sensor data. Even the same person could produce intra-class variability by performing the same activity in different ways due to external factors (e.g., surrounding environment, positions of sensors, etc.).

A possible solution to alleviate the data scarcity problem is *transfer learning*, which refers to techniques that aim at extracting knowledge from a *source domain*, and using it to improve the learning of a model on a *target domain* [8]. Data from the source domain can partially compensate the scarcity of data on the target domain. In other words, by performing some specific *task* on the source domain, the model can learn information relevant to the target problem on “external” data. *Deep transfer learning*—which refers to transfer learning applied to DNNs—has in particular become widespread with the rise in popularity of DNNs. Typically, parameters (weights and biases) of a DNN pre-trained on a source domain are transferred to another compatible DNN on the target domain. Previous works have shown that the success of the gradient descent optimisation applied during the training of a DNN is heavily dependent on the initial values of its parameters [9]. Deep transfer learning is based on the assumption that, if the features learned on the source domain are also useful for the target domain, then the parameters of a DNN pre-trained on the source domain are also adequate initial parameters for a DNN on the target domain [10,11]. Once transferred, the target DNN is *fine-tuned*, i.e., retrained using the target data to adjust the transferred parameters to the problem on the target domain as needed.

While deep transfer learning has become standard for the image processing, it has not reached the same level of maturity when time-series data are involved for several reasons. Firstly, time-series data are rather scarce due to the high cost of the labelling task for a specific application. This results in a lack of very large-scale time-series dataset (like ImageNet for images). Secondly, the development of a transfer learning method working for any type of time-series data are confronted with the difficulty that data formats on the target domain can significantly vary depending on the application. Some sensors can for instance provide “sparse” time-series containing data points unevenly spaced in time indicating events, while others provide “non-sparse” time-series consisting of data values evenly spaced in time and sampled at high frequencies. Additionally, different applications of ubiquitous computing may use different numbers and types of sensors because of differences in the relevance of devices, their obtrusiveness or easiness to setup, etc. Those applications thus rely on data consisting of different numbers of *channels*, where we refer to a *channel* as one dimension of a sequence of sensor recordings. For instance, a temperature sensor provides a single channel sequence, while three-axis accelerometers record three channels, each indicating the acceleration on one axis, etc.

We propose a transfer learning method for time-series that leverages existing datasets to bypass the issue of data scarcity on the target domain. We carry out our studies using non-sparse time-series datasets because they are the most common type of data in ubiquitous computing applications. Transfer learning for images has shown that learning general image features on ImageNet led to successful transfer of information on various target domains. In a similar way, we aim at learning general features for non-sparse time-series data which could be re-purposed to various target domains. We hypothesise that learning features related to the type of time-series data could achieve this goal. We therefore propose to use *sensor modalities* as labels, which are commonly available. We consider that two sensors are part of the same modality group if they measure the same type of measurement, and perform it in similar ways. For instance, similar devices acquiring acceleration placed at different locations are part of the same sensor modality group; acceleration acquired from two different types of devices are considered as different sensor modalities (measurement processed in different ways); and acceleration and EEG are considered as different modalities (different types of measurements).

We also design our method so that it can be applied to other target domains involving any number of channels. Our method firstly decomposes data on a source domain into single-channel data, and trains a DNN called *single-channel DNN (sDNN)* for sensor-modality classification. In other words, this DNN takes single-channel data as input and predicts their sensor modalities. Then, a model called *multichannel DNN (mDNN)* [12] is built by replicating and fine-tuning the sDNN for each of channels on the target domain. This mDNN performs recognition on the target domain by fusing outputs from all channels.

To sum up, we propose a novel, general deep transfer learning method for time-series which firstly trains an sDNN as a sensor-modality classification model using single-channel data in a source domain, and then constructs an mDNN on the target domain by replicating the sDNN on each of the target data channels. Contrary to existing time-series transfer learning methods [13,14] which focused on single-channel data analysis, our approach can be applied to data with a different number of channels. Furthermore, we introduce a new wearable-based HAR dataset, called *Cognitive Village-MSBand* dataset (*CogAge*), for the recognition of 61 activities. We carry out experiments for both HAR and ER to test our method using the CogAge and DEAP [15] datasets, respectively. Our results show that our transfer learning method consistently achieves performances at least as good as the baseline not using any transfer on both of CogAge and DEAP datasets. All research contents (source and target datasets, codes, trained DNN models) are made available to help other researchers reproduce our findings (research contents available on the following repository: https://www.info.kindai.ac.jp/~shirahama/transfer/).

This paper is organised as follows: Section 2 presents an overview of related work tackling the problem of deep transfer learning. Section 3 details our transfer learning approach. Section 4 presents the experiments carried out on the CogAge dataset for HAR, while Section 5 does the same for ER. Section 6 presents a detailed analysis using the findings of the two batches of experiments. Finally, Section 7 concludes the paper and presents potential future directions.

## 2. Related Work

We firstly focus on the image processing field where transfer learning is intensively explored because our method is inspired from works carried out in this field. We then perform an overview of transfer learning methods developed for time-series, and compare our method to them and clarify its novelties. Finally, we perform a short review of existing works related to our studies in HAR and ER.

### 2.1. Deep Transfer Learning for Images

Most general deep transfer learning methods with proven effectiveness have been developed for image modalities, due to the availability of large datasets like ImageNet (more than 14 million images labelled with over 20,000 different categories). Powerful feature extractor models like AlexNet [16], VGG-net [17] and ResNet [18] were trained on subsets of the ImageNet dataset in the frame of the ImageNet Large Scale Visual Recognition Challenges (ILSVRC) and are nowadays regularly re-used and fine-tuned for more specific applications [11]. The first studies hinting at the benefits of transfer learning emerged approximately at the same time. In [3], a key aspect of the behaviour of Convolutional Neural Networks (CNNs) was highlighted by showing that each neuron of convolutional layers encodes a specific feature, whose specificity increases with the depth of the layer using a variant of AlexNet [16]. The authors also analysed the generality of features learned by the model trained on the source domain (ImageNet) by checking its transferability on three smaller target domains. The major performance improvements showed the potential of parameter-based transfers for DNNs. In a similar fashion, Donahue et al. [19] managed to show how AlexNet could improve the performances of various target problems such as domain adaptation, object recognition, sub-category, and scene recognition. The authors of [20] trained a variant of AlexNet for image classification, and transferred it for object detection and localisation tasks, obtaining state-of-the-art results in both setups. In [21], the authors extracted features from warped regions of images, by pre-training a variant of AlexNet on a subset of ImageNet. It was then fine-tuned using the warped images as inputs for image classification on two different target domains (PASCAL VOC and a different subset of ImageNet). The transferred model was able to significantly outperform the previously best solutions on both target domains. Similarly, Oquab et al. [22] presented a study in which the layers of AlexNet trained discriminatively on ImageNet were transferred to a DNN model designed for object and action classification on the PASCAL VOC dataset. In [10], researchers analysed the impact of different transfer learning parameters for AlexNet such as number of transferred layers, using fine-tuning or not, using different subsets of ImageNet as source and target. They showed that the target performance drops when only transferring deeper layers (which were shown to encode features more specific to the source problem [3]), and how important fine-tuning on the target domain was. In addition, it was demonstrated that the transfer learning process could boost the generalisation capacity of the network compared to not using it.

More recently, diverse attempts to further improve the efficiency of transfer learning by changing different parameters have been made. In [23], the authors present a method based on information theory to automatically find the most suitable source domain to perform transfer given a target domain with a specific task. Assuming that different CNNs with similar architectures have each been trained on a source domain, they propose a ranking metric called “transferability” by computing the Mutual Information between the target labels and the features of each of the CNNs. The transferability can be used to estimate how much a specific source domain can reduce the uncertainty in predicting the test labels. Experiments showed that the top ranked CNNs in terms of transferability led to the best performances after transfer and fine-tuning on the target domain. In [24], a method to improve the fine-tuning procedure on the target domain is presented. Assuming that a pre-trained model is available on a source domain (e.g., ImageNet), the authors propose to jointly train a “policy network” using a Gumbel Softmax distribution and a DNN for the target classification task. For each testing image and layer of the target DNN, the policy network is used to determine whether the weights of the layer should be frozen or fine-tuned using the image. Experiments showed that the proposed adaptive fine-tuning approach led to better results than other state-of-the-art fine-tuning and regularisation techniques. On a similar idea, Li et al. [25] investigate the effectiveness of different regularisation approaches whose aim is to keep the weights which are fine-tuned on the target domain as close as possible to those learned on the source domain. A baseline consisting of a regular fine-tuning of the target DNN was also tested. Experiments for image classification and segmentation showed that all regularisation approaches led to better performances than the baseline.

It can be noted that all aforementioned works are based on supervised pre-training of one or several DNN models on a source domain. Unsupervised pre-training using unlabelled data has also been attempted for image modalities [11,26], but failed to yield performances as good as supervised pre-training regardless of the quantity of available unlabelled data. This highlights the superiority of using labelled data on the source domain, and motivates our choice to define a supervised pre-training using sensor modalities as labels.

### 2.2. Deep Transfer Learning for Time-Series

Transfer learning techniques have been much less explored for time-series data because of the scarcity of data in the ubiquitous computing field, and the absence of a large-scale labelled dataset like ImageNet. Nevertheless, past works have attempted to tackle this issue with different degrees of generality. On a general level, [27] defines different types of transfers that can be applied to wearable-based HAR. It introduces the concepts of instance transfer that re-uses data in the source domain to train a model in the target domain, feature representation transfer that finds a feature mapping between the source and target domains, and parameter transfer that transfers parameters from a model trained on the source domain to model for the target domain. On a more specific level, several works presented results of parameter transfer for HAR. In [28], the results in several scenarios of parameter transfer such as transfer between subjects, datasets, sensor localisation, or modalities were presented. All transferred models were tested against a baseline that “regularly” trains the model on the target domain only. Despite poor relative performances of the transferred models compared to the baseline, the study highlighted some interesting phenomena. The performances of transfers were sensibly better when parameters of the lower layers were transferred. In [29], a transfer approach for CNN when labelled target data are scarce but labelled source data are available was presented. It firstly trains a CNN using labelled data on the source domain and defines a CNN with similar architecture on the target domain. The target CNN is then trained on unlabelled data to minimise the distance between its parameters and the ones of the source CNN. It, however, only works under the assumption that the set of activities on the source and target domains is the same. In [30], an iterative co-training approach using classification models trained on labelled source data to attribute pseudo-labels to unlabelled target data was presented. It works under the assumption that source and target domains contain the same labels. A transformation which minimises the maximum mean discrepancy between labelled and pseudo labelled examples is found. Source and target data are then projected into a common space using the transformation, and classifiers are trained on the projected data to attribute more reliable labels.

However, the scope of the above-mentioned studies [28,29,30] is limited to a specific application field (wearable-based HAR), and by strict conditions on the similarity between the source and target domains (e.g., same set of labels, same type of data, etc.). Compared to this, our transfer learning method can be generally applied to any application field using time-series data. This generalisation is demonstrated by targeting wearable-based HAR in Section 4 and ER in Section 5. In addition, our method does not require source and target domains to be characterised by the same label or data types.

To our best knowledge, only two past works proposed a general transfer learning method that is potentially usable for different ubiquitous computing applications. In [14], a Recurrent Neural Network (RNN) was trained using data from the UCR Time Series Classification Archive (UCRTSCA) [31] that consists of 85 small-scale univariate time-series datasets covering a wide range of sensor modalities, such as accelerometer data, energy demand, chemical concentration in water, etc. The RNN composed of an encoder and decoder was trained to reproduce its input on its output layer using a subset of 24 datasets of the UCRTSCA (source domain). After this pre-training step, the encoder was used as a feature extractor for a Support Vector Machine (SVM) fine-tuned on each of 30 other datasets of the UCRTSCA (target domain). The experimental results indicated that data on source domains not necessarily related to the target domain were still useful for achieving state-of-the-art results. In [13], a method to compute the similarity between source and target datasets to determine the most suitable dataset for transfer was proposed. It assumes that one labelled target and several labelled source datasets are available. For each dataset, the method firstly computes the average of sequences for each class. The barycentre of all class averages is then computed to yield a “characteristic sequence” of the dataset. The similarity between two datasets is computed using the Dynamic Time Warping (DTW) distance between their respective characteristic sequences. The source dataset with the lowest distance is then chosen and used to train a DNN. Its weights are finally transferred on the target domain for fine-tuning. Experiments carried out on the 85 datasets of the UCRTSCA showed that the transfer yielded better classification performances when the similarity between source and target was higher.

However, the methods in [13,14] remain limited to the case of processing single-channel sequences since their experiments were both carried out on the UCRTSCA, and do not present how to generalise it to multichannel sequences. In contrast, we propose a multichannel DNN architecture that can be widely used for multichannel sequences in different ubiquitous computing applications.

### 2.3. Sensor-Based HAR and ER

*Sensor-based HAR:* HAR is one of the most popular research topics of ubiquitous computing due cheap and widespread motion sensors such as accelerometers and gyroscopes, the relative simplicity to acquire labelled data compared to other applications, and its potential applications in several domains such as assistive living, surveillance, improvement of quality of life, or gaming [1,2]. We mainly focus on deep-learning used for sensor-based HAR (i.e., HAR using low-level readings under the format of time-series provided by wearable sensors) as opposed to video-based HAR (i.e., relying on vision sensors) [27,32].

Growing evidence from past studies on sensor-based HAR has shown that DNNs could successfully be used for sensor-based HAR using continuous time-series data acquired from wearable sensors [1,4,5], and outperformed traditional approaches relying on manual crafting of features [2]. Recent works have in particular highlighted the importance of convolutional-based DNN architectures [1,2,4], recurrent architectures involving LSTM cells [5], or hybrid models combining both convolutional and LSTM layers [2,4,5] in obtaining state-of-the-art performances on various HAR benchmarks.

*Sensor-based ER:* ER is an important component of Affective Computing which designates the study of techniques teaching machines to automatically recognise the human effect to enhance computer–human interactions [33]. This goal is usually reached by using machine learning techniques on data acquired by sensors and labelled with emotion annotations. ER is approached differently depending on the type of sensor modality used to acquire the data. A large part of the ER literature over the past decades has focused on the analysis of facial expressions in RGB images and/or videos, or speech in audio signals [34]. However, audiovisual sensor modalities are not always available due to difficulties to setup properly the cameras in real-life, concerns about privacy or use-case scenarios where parts of the subjects’ faces are hidden [35]. As a consequence, interest in *sensor-based ER*—the study of ER using wearable sensors recording physiological signals (e.g., Electro-encephalography (EEG), Electrodermal Activity (EDA), Electroocculography (EOG), Electromyography (EMG), etc.)—has grown with the increasing availability of wearable devices.

One of the first proposals for a sensor-based ER system can be found in [36]. In this study, the authors proposed to use the EEG channels of the DEAP dataset for the two-class classification problems of low versus high arousal/valence/dominance/liking using the 1-minute data records. Hand-crafted features were firstly computed on the power spectrum of overlapping segments of the original 1-min signals, then projected using a non-parametric model based on the k-Nearest Neighbour (kNN) approach to provide a feature vector for the 1-minute record. A 1NN classifier provided classification performances in a subject-independent setup. With the increasing popularity of deep-learning, researchers have also tried to apply DNNs to sensor-based ER. The authors of [37] proposed an approach computing hand-crafted features on the power spectrum of the DEAP EEG signals and sending them to a stacked autoencoder based on MLP. Classification results for three classes of arousal/valence (low/medium/high) were provided in a subject-independent context. In [7], a bi-modal deep autoencoder approach was proposed to learn features from EEG and EOG signals in an unsupervised way and provide classification results in a subject-dependent context on both DEAP and SEED datasets. In [38], a residual multimodal LSTM architecture using one residual LSTM network to learn features from each input sensor channel was proposed. Classification results for the binary classification of arousal and valence on the DEAP dataset in a subject-dependent context were provided.

It should be noted that all aforementioned works in Section 2.3 have focused on sensor-based HAR or ER without transfer learning. In contrast, we propose a time-series transfer learning approach and test its applicability in both HAR and ER contexts.

## 3. Methodology Description

Using the notations of [8,27], we define a *labelled domain dataset* D as a combination of two components: one set of data instances X and a vector of associated labels Y. A *task*T is defined as the association of Y with a *predictive functionf* to be learned from the labelled data. The source and target domains datasets are referred to as $D_{S}=\left\{X_{S},Y_{S}\right\}$ and $D_{T}=\left\{X_{T},Y_{T}\right\}$, while the source and target tasks are denoted by $T_{S}=\left\{Y_{S},f_{S}\right\}$ and $T_{T}=\left\{Y_{T},f_{T}\right\}$, respectively. We assume that $T_{T}$ and $D_{T}$—which respectively represent the target ubiquitous computing problem to solve and its associated labelled dataset—are available.

**Figure 1:**
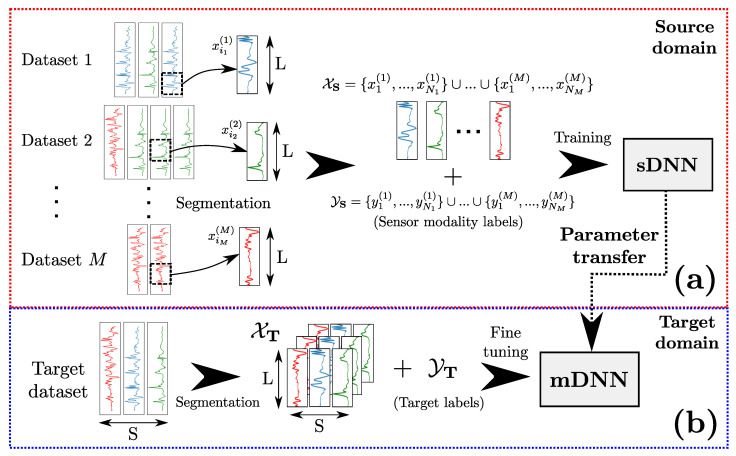
An overview of our transfer learning method. (**a**) A labelled source dataset of single-channel sequences $\left(X_{S},Y_{S}\right)$ is created by collecting segments $x_{i}^{\left(j\right)}$ of length *L* from *M* datasets and attributing them sensor modality labels $y_{i}^{\left(j\right)}$. $\left(X_{S},Y_{S}\right)$ is then used to train a sDNN that predicts the sensor modality of each segment. (**b**) A mDNN is built to learn the predictive target function $f_{T}$. The weights of the trained sDNN are transferred to the mDNN. The latter is then fine-tuned on the target domain using $\left(X_{T},Y_{T}\right)$.

**Figure 2:**
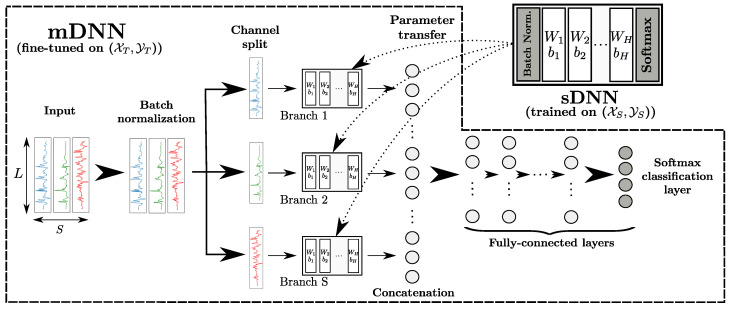
mDNN used for the learning of $f_{T}$ on the target domain. The input segments of the target dataset $X_{T}$ are sent through a batch normalisation layer. All sensor channels are then separated and processed by *S* branches with the same number and type of hidden layers as the sDNN trained on the source dataset $\left(X_{S},Y_{S}\right)$. The outputs of the *S* branches are concatenated and sent through fully-connected and softmax layers for classification. The mDNN is fine-tuned using the target dataset $\left(X_{T},Y_{T}\right)$.

We propose a *deep transfer learning* strategy based on transferring DNN weights learned on a sensor-modality classification problem on $D_{S}$ to another DNN trained to solve $T_{T}$ on $D_{T}$. Our method—illustrated in Figure 1—belongs to the category of *inductive transfers*, since the source and target tasks are different ($T_{S}\neq T_{T}$). It consists of the following steps:

1. **Definition of $D_{S}$ and $T_{S}$:**$X_{S}$ is firstly built by considering *M* multichannel time-series datasets. Every multichannel sequence in the $j^{th}$ dataset (1⩽j⩽M) is decomposed into individual channels, each of which is divided into *segments* of length *L* using a sliding window approach. The segments are aggregated to form the source dataset $X_{S}$ defined in Equation (1):
  (1) $$X_{S}=\overset{M}{\underset{j=1}{⋃}}\left\{x_{i}^{\left(j\right)}\in R^{L}\;|\;1⩽i⩽N_{j}\right\}$$
  where $x_{i}^{\left(j\right)}$ refers to the $i^{th}$ segment of the $j^{th}$ source dataset, and $N_{j}$ is the total number of segments obtained from the $j^{th}$ source dataset. In other words, $X_{S}$ is the union of all segments extracted from the *M* source datasets. The source task $T_{S}$ is defined as the classification of sensor modalities on $D_{S}$. Sensor modality labels $Y_{S}$ are defined by the following Equation (2):
  (2) $$Y_{S}=\overset{M}{\underset{j=1}{⋃}}\left\{y_{i}^{\left(j\right)}\in \left\{1,2,\ldots ,C_{S}\right\}\;|\;1⩽i⩽N_{j}\right\}$$
  where $C_{S}$ is the number of sensor modalities (i.e., classes) of the source domain, and $y_{i}^{\left(j\right)}$ indicates the sensor modality of $x_{i}^{\left(j\right)}\in X_{S}$. $f_{S}$ is the function which attributes each $x_{i}^{\left(j\right)}$ to its corresponding sensor modality $y_{i}^{\left(j\right)}$.
2. **Learning of $f_{S}$:** A single-channel DNN (sDNN) is used to learn $f_{S}$, as shown in Figure 1a. For the sDNN architecture, a batch normalisation layer used to perform a regularisation on the segments in $X_{S}$ to address the issue of the heterogeneity of the source data. Assuming the sDNN contains $H\in N^{*}$ hidden layers, we denote the weight matrix and bias vector of the $k^{th}$ layer (1⩽k⩽H) as $W_{k}$ and $b_{k}$, respectively. Finally, a softmax layer with $C_{S}$ neurons is added, with each neuron of the layer outputting a value which is an estimation of probability to its corresponding class. This way, the sDNN can classify the segments of $X_{S}$ using the labels $Y_{S}$.
3. **Initialisation of a multichannel DNN (mDNN):** A mDNN is defined to learn $f_{T}$, as shown in Figure 2. It is trained using $X_{T}$ which contains multichannel segments $X\in R^{L\times S}$, with *S* being the number of channels of the target dataset and $Y_{T}$ which contains associated labels $Y\in \{1,2,\ldots ,C_{T}\}$ with $C_{T}$ being the number of classes of the target problem. For the mDNN architecture, a batch normalisation layer is applied to the segments to perform an operation akin to a standard normalisation on the input of the network. The *S* sensor channels are then separated. The $s^{th}$ sensor channel (1⩽s⩽S) is processed by an ensemble of hidden layers of the same number and type as the hidden layers of the sDNN. We refer to this ensemble of layers as a *branch* of the mDNN, as depicted in Figure 2. The output of each branch is then concatenated and connected to fully-connected layers. A softmax layer with $C_{T}$ neurons is then added to output class probabilities for the $C_{T}$ target classes.
4. **Transfer of weights from the sDNN to the mDNN:** The weights $W_{k}$ and biases $b_{k}$ of the *H* hidden layers of the sDNN learned on $\left\{D_{S},T_{S}\right\}$ (not including batch normalisation and softmax layers) are transferred to the branches of the mDNN, as shown in Figure 2. In other words, the $k^{th}$ layer of the $s^{th}$ branch (for 1⩽k⩽H and 1⩽s⩽S) has its weight and bias matrices $W_{k}^{\left(s\right)}$ and $b_{k}^{\left(s\right)}$ initialised as $W_{k}$ and $b_{k}$, respectively.
5. **Learning of $f_{T}$:** The mDNN is fined-tuned using $\left(X_{T},Y_{T}\right)$ to learn $f_{T}$, which is the predictive function for the target ubiquitous computing problem.

In our experiments, we used CNNs as sDNNs and the branches of a mDNN because of their good performances for time-series classification in diverse application fields of ubiquitous computing [13]. We therefore refer to our transfer approach from now on as *CNN-transfer*. We also tested fully-connected and recurrent layers with Long-Short-Term-Memory (LSTM) cells for hidden layers of the sDNNs. However, both of them ended up performing worse than convolutional layers in all configurations. Those results are consistent with past works which showed that CNNs are better feature extractors than fully-connected or LSTM networks in a time-series classification context [39]. For LSTM-based architectures in particular, finding a properly performing baseline architecture (i.e., not using any transfer) on the target datasets ended up being impractical. The high number of LSTM parameters and the large size of our multichannel architecture limited the complexity of the tested mDNN. In addition, using multichannel data segments with long temporal length significantly extended the training time of LSTM models (based on the backpropagation-through-time algorithm) compared to CNN-based approaches (even in configurations where simple LSTM architectures were tested), and increased the likelihood to overfit. Both phenomena were already highlighted in past HAR literature [5] and comforted our decision to use CNNs in our experiments. Results obtained by the LSTM architectures we tested are uploaded on our repository (link provided in the Supplementary Materials).

Four datasets taken from the UCI machine learning repository [40] were used in our study to build the source domain, covering 16 different sensor modalities in total: *OPPORTUNITY* [41] (accelerometers and IMUs data for Activities of Daily Life recognition), *gas-mixture* [42] (gas concentration and conductance chemical sensor readings data), *EEG-eye-state* [43] (ElectroEncophaloGraphy (EEG) data for open/close eye recognition) and *energy-appliance* [44] (data from a low-energy house such as temperature, humidity, air pressure, and energy consumption for the prediction of energy consumption). $C_{S}=16$ sensor modalities were obtained in total by using the documentation and information provided by the authors of each dataset. A sDNN trained on the source domain therefore had a softmax layer with 16 units, each outputting the probability that a segment belongs to one sensor modality. The complete list of modalities in the source domain is provided in Table 1.

It should be noted that OPPORTUNITY and gas-mixture are notably larger than the other datasets. The question of balancing the source datasets therefore arose. We tested two approaches: one downsampling the largest dataset so that all datasets provide a balanced contribution, the other taking as much data as possible from each dataset. Both approaches yielded comparable performances, in accordance with a similar analysis where the quantity of data to train transferred models is changed [11]. We report in the following discussions the best performances attained by the aforementioned two approaches.

## 4. Experiments for Wearable-Based Human Activity Recognition

In this section, we introduce the *Cognitive Village-MSBand* dataset for wearable-based HAR—referred to as *CogAge dataset* for the sake of simplicity—and the results of the experiments carried out on it.

### 4.1. Dataset Description

The CogAge dataset was built by considering human activities as a series of simpler actions, referred to as *atomic activities*. It aggregates the data from four subjects performing a total of 61 different atomic activities split into two distinct categories: six *state activities* characterising the pose of a subject, and 55 *behavioral activities* characterising his/her behavior. The complete list of activities is provided in Table 2. It can be noted that a behavioral activity can be performed while being in a particular state (e.g., drinking can be performed either while sitting or standing). Because this overlap between state and behavioral activities could potentially prevent a proper definition of classes (e.g., drinking while sitting could either be classified as drinking or sitting), two classification problems were considered, one considering exclusively the six state activities, the other the 55 behavioral activities.

All four subjects were asked to wear three different devices during the data acquisition process:

- Google NEXUS 5X smartphone placed in a subject’s front left pocket, providing five different sensor modalities: three-axis accelerometer, gravity sensor, gyroscope, linear accelerometer (all sampled at 200 Hz) and magnetometer (50 Hz).
- Microsoft Band 2 placed on a subject’s left arm, providing two different sensor modalities: three-axis accelerometer and gyroscope (67 Hz).
- JINS MEME glasses placed on the subjects’ head, providing five different sensor modalities: three-axis accelerometer and gyroscope (20 Hz), blink speed, strength measurements, and eye-movement measurements (all discrete signals indicating an event).

All four subjects took part in two data acquisition sessions (#1 and #2) where each of 61 atomic activities is executed at least 20 times, and each execution lasts for 5 s. Because the smartwatch was placed on the left arm, the choice of the arm performing some behavioral atomic actions indicated with a * in Table 2 may impact the recognition performances. Two different datasets were therefore created for the behavioral classification problem: one gathering executions only performed by the left hand, the other gathering executions performed indifferently by the left or right hand. We refer to the former and the latter as *Behavioral Left-Hand-Only (BLHO)* and *Behavioral Both-Hands (BBH)* datasets, respectively. To build the training and testing sets, we followed two strategies: one splitting the data using a subject-dependent setup where data from the same subjects are included in the training and testing sets, the other using a subject-independent split where distinct subsets of subjects provide data for the training and testing sets. The subject-dependent classification problem has a higher simplicity, while the subject-independent one is more representative of real use-cases. For the subject-dependent split, the data from session #1 were used as a training set, while those from session #2 as a testing set. The total number of executions of each dataset is summarised in Table 3. For the subject-independent setup, a leave-one-subject-out cross validation was performed: the data from one subject in both sessions #1 and #2 were used as testing set, and the data from the three other subjects as training set. All four subjects were used as testing subject once. The number of executions per subject is provided in Table 4.

### 4.2. Experimental Setup

Because of their different nature (data characterised by spikes instead of continuous values), the blink speed, strength, and eye-movement signals of the JINS glasses were not used in this study. In addition, preliminary experiments using all devices showed that the smartphone magnetometer had little impact on the final classification performances. Our baseline study therefore used the smartphone accelerometer, gyroscope, gravity sensor, linear accelerometer, the data from the smartwatch accelerometer and gyroscope, and the data from the JINS glasses accelerometer and gyroscope.

**Figure 3:**
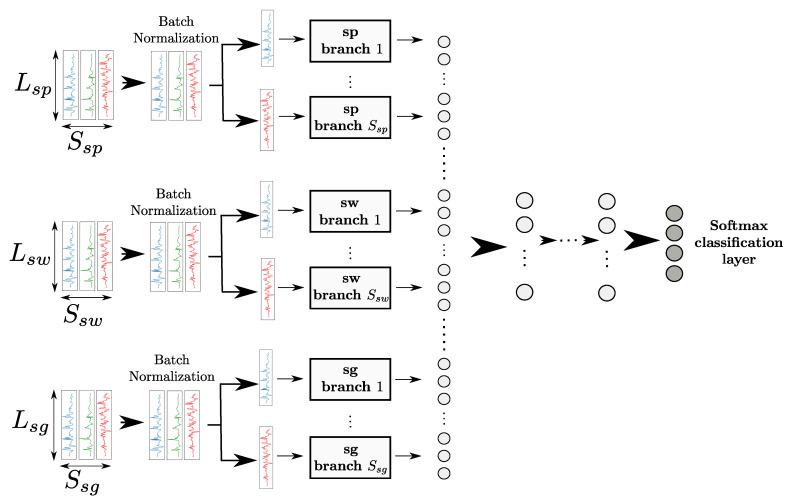
Model used on the CogAge dataset. Each of the three mDNNs processes the smartphone (sp), smartwatch (sw) or smartglasses (sg) data. $L_{*}$ and $S_{*}$ with ∗∈{sp,sw,sg} refer to the segment length and number of sensor channels, respectively. Outputs from the three mDNNs are concatenated and fed into fully-connected and softmax layers.

The differences in sampling frequencies of those sensors affect the size of the 5-s segments, and the shape of the input of our DNN models. To take this into account, we define three different sDNNs processing data coming from the smartphone, smartwatch, and smartglasses, respectively. One sDNN is associated with all channels generated from one of the three devices, as shown in Figure 3. The outputs of all sDNNs are then concatenated and fed into fully-connected and softmax layers, as shown in Figure 3.

Because of data transmission problems, all channels do not necessarily have a length of exactly 5 s. We therefore decided to use the first 4 s of each record. This leads to segments of shape $L_{sp}\times S_{sp}=800\times 12$, $L_{sw}\times S_{sw}=267\times 6$ and $L_{sg}\times S_{sg}=80\times 6$ for the smartphone, smartwatch, and JINS glasses, respectively. For our CNN-transfer approach, three sDNNs are trained separately for sensor modality classification on the source domain, each taking input of sizes $L_{sp}$, $L_{sw}$ and $L_{sg}$, respectively. The resulting mDNN comprises S=12+6+6=24 branches. The weights of each sDNN are then transferred to the mDNN of one device. As indicated in Section 3, we use the OPPORTUNITY [41], gas-mixture [42], EEG-eye-state [43] and energy-appliance [44] datasets to build the source domain. For comparison, we report the performances of the following two approaches:

- *Train on Target Only (TTO):* Baseline approach which only trains a mDNN on the target domain, without using transfer learning. The weights of the mDNN are initialised using a Glorot uniform initialisation [9].
- *Variational Autoencoder-Transfer* (*VAE-transfer*): Approach which trains a sDNN on the source domain in an unsupervised way. The sDNN to be transferred is considered as the encoder part of a convolutional Variational Autoencoder (VAE) [45]. The encoder of a VAE learns the parameters of a Gaussian probability density characterising a compressed representation of the input in a lower dimensional space called *embedding space*. A sample is then drawn from such learned Gaussian distribution and sent as input of a decoder—DNN whose structure mirrors the encoder—which is trained to reconstruct the encoder input on its output layer. The ensemble encoder–decoder is trained to reproduce the segments of the source domain as accurately as possible. The weights of the encoder are then transferred to a mDNN. For the CogAge dataset in particular, three VAEs taking input of sizes $L_{sp}$, $L_{sw}$ and $L_{sg}$, respectively, are trained and transferred.

**Figure 4:**
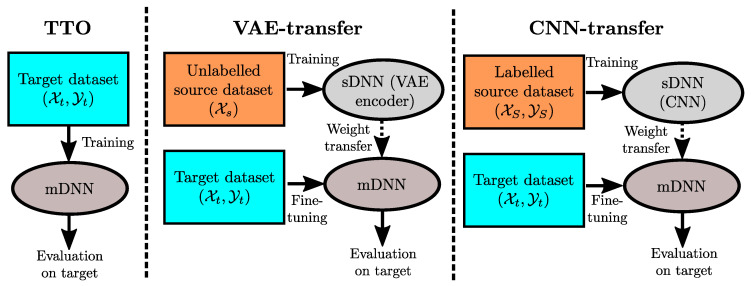
Flowchart of the three approaches tested on the CogAge dataset: TTO (no transfer), VAE-transfer, and CNN-transfer. The mDNN follows the architecture described in Figure 3.

We also tested a third approach which transfers weights from the source domain without performing any fine-tuning on the target domain. It, however, yielded performances significantly worse than all other methods. We thus decided not to report the results of this approach. The three aforementioned approaches tested on the CogAge dataset are summarised in Figure 4.

The hyper-parameters of the mDNN were firstly optimised by trial and error for TTO. Both CNN-transfer and VAE-transfer were then performed by re-using the same parameters. All DNN parameters are provided on our repository whose link is provided in the Supplementary Materials of this paper. For CNN-transfer, all sDNNs were trained for 25 epochs using the ADADELTA optimiser [46] with a categorical cross-entropy loss function. For VAE-transfer, the encoder–decoder ensemble was trained for 10 epochs using an ADADELTA optimiser. A Mean Square Error reconstruction term regularised by the Kullback–Leibler divergence between the Gaussian distribution learned by the encoder and the Standard Normal Distribution was used as loss function. In addition, 90% of the source data were used as training set. The remaining 10% were used as a validation set to validate the sDNN parameters. In the case of TTO, the weights of the mDNN were initialised using the Glorot uniform initialisation. The mDNN was then fine-tuned for the three classification problems—state, BLHO and BBH—using the ADADELTA optimiser with a categorical cross-entropy loss function for 150 epochs. All models were coded using the Keras library (version 2.4.2) with Tensorflow backend(version 1.12), and trained using a 16 GB RAM machine with an Intel i7-7700K CPU and a Nvidia GTX 1080Ti GPU.

The accuracy, the average F1-score (AF1), and Mean Average Precision (MAP) were used as evaluation metrics. The MAP is based on the computation of class Average Precisions (APs). For each class, test examples are ordered by decreasing probabilities provided by the softmax layer of a mDNN. Precision is then computed at each position of an example of the class in the ordered list. Those precisions are then averaged to compute the AP of the class, and the class APs averaged to yield the MAP. Because of potential overlapping between state and behavioural activities, AP is a convenient metric for examining whether an execution was preferentially classified into the most relevant class or not.

### 4.3. Results

The results of the three classification problems are provided in Table 5 for the subject-dependent configuration and Table 6 for the subject-independent one. The main observations for both setups can be highlighted as follows:

- The results of the state classification problem are relatively uniform across our transfer and the baseline approaches. We attribute this to two factors. Firstly, the state classification problem is significantly simpler than BBH or BLHO because it contains a low number of fairly distinct classes. Secondly, the fairly small size of the testing set which makes a few misclassified examples result in a drop of a few percent(s) in evaluation metrics. With these factors in mind, it can be observed that CNN-transfer, TTO and VAE-transfer all return predictions on the testing set differ only on a few examples.
- For behavioural activity classification, VAE-transfer performs mediocre overall, and ends up yielding results worse than both CNN-transfer and TTO.
- Our transfer approach consistently yields better results than TTO for both BLHO and BBH classification problems. It can be noted that CNN-transfer provides better performances than TTO for all test subjects in the subject-independent configuration.

A detailed analysis of class APs for behavioural activities was carried out in the subject-dependent configuration to examine whether some transfer setups could benefit particular activities or not. Class AP plots are uploaded on our repository (link provided in the Supplementary Materials of this paper). We could observe the superiority of CNN-transfer by computing some global statistics on all activities. CNN-transfer yielded better class APs than TTO for 40/55 and 42/55 behavioral activities for BLHO and BBH, respectively. CNN-transfer obtained an 8.59% and 8.27% improvement in AP compared to TTO for the BLHO and BBH classification, respectively. For others’ activities, CNN-transfer underperformed TTO by smaller margins, with an average AP gap of 3.07% and 3.54% for the BLHO and BBH classification, respectively. The overall results suggest that CNN-transfer allows for obtaining performance improvements compared to TTO, but it is difficult to designate activities which specifically benefit from the transfer. We could in particular check that activities similar to those contained in the OPPORTUNITY dataset (which is part of the source domain, and contains data about opening and closing doors/drawers) were not always yielding better results compared to TTO.

## 5. Experiments for Wearable-Based Emotion Recognition

This section presents the results of our transfer learning approach for ER. The experiments are conducted on a popular benchmark dataset for wearable-based ER: DEAP [15].

### 5.1. Dataset Description

The DEAP dataset aggregates data from 32 subjects who watched 40 one-minute-long music videos, selected to induce a wide range of emotions. During the experiments, each subject was wearing on his/her head a sensor equipment yielding a total of 40 sensor channels (S=40): 32 EEG channels and eight channels returning peripheral physiological signals (EOG, EMG, GSR, BVP, temperature, and respiration). The labeling was performed using the Circumplex model which decomposes emotions along two main axes: arousal (level of excitement) and valence (level of pleasantness). Each subject was asked after each visualisation to rate his/her level of arousal and valence on a 9-point scale from 1 (very low) to 9 (very high). We used the pre-processed version of the dataset, with all 40 channels downsampled to a frequency of 128 Hz.

### 5.2. Experimental Setup

To evaluate data labelled using the Circumplex model, numerous studies defined emotion recognition either as a 2-class problem between low (<5) and high (⩾5) arousal/valence, or 3-class problem between low (<3), medium (⩾3 and <6), and high (⩾6). Sensor-based ER is still a relatively immature research topic due to its inherent difficulty caused by several factors such as challenges to get properly labelled data, the high intra-class variability when using physiological signals, etc. As a result, a large part of the ER literature performed experiments in a subject-dependent context, while the few subject-independent studies could only report mediocre classification results [37,47]. We therefore decided to use the subject-dependent setup of [7] in which the authors trained a bi-modal autoencoder processing both EEG and other modalities and taking non-overlapping segments of 1 s (L=128) as inputs for a 2-class classification problem for arousal and valence. The data segments from all 32 subjects of the DEAP dataset were mixed and evenly split into folds for a 10-fold cross-validation. In our experiments, we train two mDNNs with S=40 branches, one for arousal and the other for valence classification. Both are evaluated using the classification accuracy as an evaluation metric.

Similarly to the CogAge dataset, we test the performances of the TTO, VAE-transfer, and CNN-transfer approaches on DEAP, which are defined in Section 4.2. The parameters of the mDNN were firstly optimised for TTO by trial and error, and then re-used for both CNN-transfer and VAE-transfer. All DNN parameters are provided on our repository whose link is provided in the Supplementary Materials of this paper. For CNN-transfer and each of arousal and valence classifications, an sDNN was trained for 100 epochs using the ADADELTA optimiser with a categorical cross-entropy loss function. For VAE-transfer, the same training setup as described in Section 4.2 for the CogAge dataset is used. In addition, 90% of the source data were used as a training set. The remaining 10% were used as validation set to validate the sDNN parameters. Weights of the sDNN were transferred to construct a mDNN on the target domain. The rest of the weights of the mDNN were initialised using the Glorot uniform initialisation [9]. Two mDNNs—one for arousal and the other for valence—were then fine-tuned for each fold using the ADADELTA optimiser with a categorical cross-entropy loss function for 300 epochs.

### 5.3. Results

The results for arousal and valence classification on the DEAP dataset are summarised in Table 7 and Table 8, respectively. Similarly to the CogAge dataset, VAE-transfer was again outperformed by both CNN-transfer and TTO. CNN-transfer consistently outperformed TTO on all 10 folds for both arousal and valence classification problems. This validates the effectiveness of our transfer approach. In addition, our method yielded significantly better results than those obtained in [7] using bi-modal AEs.

## 6. Analysis

The experiments on both CogAge and DEAP datasets showed that the best performances were obtained by our transfer method based on supervised pre-training using sensor modality labels. Our transfer approach is based on the assumption that it could help in cases where labelled training data on the target dataset are scarce. In order to further check this assumption, we carried out additional experiments with reduced amounts of training data on both CogAge (subject-dependent configuration) and DEAP datasets. On both target datasets, we randomly downsampled the training dataset to 5, 25, 50, and 75% of its original size while keeping the same number of testing examples, and compute the classification performances on the same number of testing examples. Table 9, Table 10 and Table 11 show the results for State/BLHO/BBH classification on CogAge, arousal classification on DEAP, and valence classification on DEAP, respectively.

The main observation is that CNN-transfer keeps outperforming TTO at all levels of downsampling of the target training sets. CNN-transfer yields a consistent improvement for both BLHO and BBH problems compared to TTO at all downsampling levels. Performances on the state classification remain relatively uniform between all three tested methods in most configurations due to the highest simplicity of the problem. Larger differences in performances can be observed in the case where the training set was downsampled the most (5%). In that configuration, CNN-transfer clearly outperforms the two other approaches which indicates its effectiveness in configurations with few training examples. The same consistency can be observed on the DEAP dataset as CNN-transfer also outperformed TTO on all 10 folds of the dataset. VAE-transfer remains outperformed by both TTO and CNN-transfer in most tested configurations.

To obtain a better idea of the reasons behind the performance improvements of our transfer approach compared to the case without transfer, we performed a low-level analysis on neurons of an mDNN to identify differences between TTO and CNN-transfer. On both CogAge (subject-dependent setup) and DEAP, TTO and CNN-transfer were respectively used to train two mDNNs with the same architecture (shown in Figure 3). For each neuron or layer of this architecture, we can compare metrics computed on the mDNN trained with TTO to those on the mDNN trained with CNN-transfer. This way, we can find in which neurons and layers the biggest differences (or similarities) can be found between TTO and CNN-transfer.

Given two trained mDNNs—one using TTO and the other using CNN-transfer—we computed an importance score for each neuron which indicates its relevance (and the one of the feature it encodes) to the target classification problem. For this, we used the *Neuron Importance Score Propagation (NISP)* [48] and *Infinite Feature Selection (InfFS)* [49] methods. NISP can be applied to any DNN involving fully-connected, convolutional or pooling layers. It is a score backpropagation method which assumes that importance scores are available for the neurons on the penultimate layer (i.e., last one before the softmax layer). Those scores can be obtained by any feature ranking approach. Similarly to [48], we chose the InfFS method to compute them, mainly since it showed its effectiveness for DNN architectures involving convolutional layers. NISP then backpropagates the InfFS scores to the prior layers so that an importance score can be attributed to each neuron of the DNN. Let $n^{\left(k\right)}$ be the number of neurons of the kth layer, $s_{i}^{\left(k\right)}$ be the importance score of the ith neuron of the kth layer ($1⩽k⩽n^{\left(k\right)}$), $w_{ij}^{\left(k\right)}$ be the weight connecting the ith neuron of the (k−1)th layer to the jth neuron of the kth layer ($1⩽i⩽n^{\left(k-1\right)}$ and $1⩽j⩽n^{\left(k\right)}$). Then, $s_{i}^{\left(k\right)}$ is computed as

(3) $$s_{i}^{\left(k\right)}=\sum_{j=1}^{n^{\left(k+1\right)}}\left|w_{ij}^{\left(k\right)}\right|s_{j}^{\left(k+1\right)}$$

In other words, the importance score of a neuron in a layer is the sum of the scores of all neurons of the next layer that it has a connection with, weighted by the absolute value of the neural weights. This formula can be used to backpropagate importance scores in fully-connected layers. How to apply it to convolutional and pooling layers can be found in the supplementary materials of [48].

**Figure 5:**
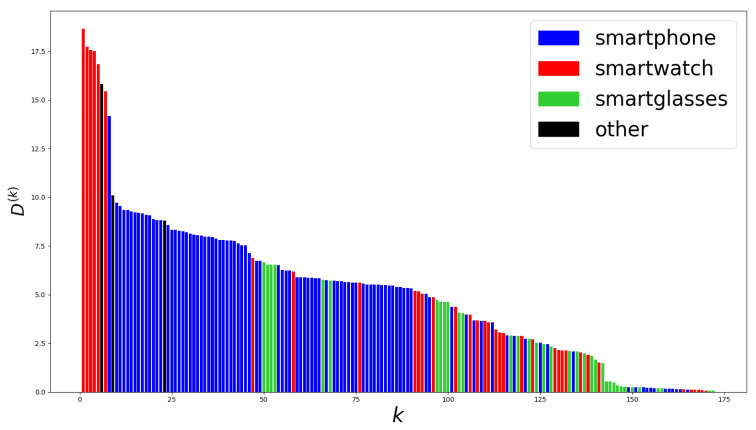
Layer differences $D^{\left(k\right)}$ for all layers of mDNNs trained using TTO and CNN-transfer for BBH classification on the CogAge dataset. Each bar corresponds to a layer and represents its difference between TTO and CNN-transfer. Layer differences are arranged in decreasing order. For each of them, we indicate if it was computed for a layer belonging to a branch processing smartphone, smartwatch, or smartglasses data. Layers not belonging to any branch (e.g., concatenation or fully-connected layers) are categorised as “other”.

The study was carried out for BBH classification on the CogAge dataset, as we thought that the largest performance gap between TTO and CNN-transfer would lead to the clearest differences between their neuron importance scores (we obtained similar results for BLHO classification on the CogAge dataset; associated plots are provided on our repository whose link is provided in the Supplementary Materials). Using NISP and InfFS, we got vectors of neuron importance scores for all layers of both mDNNs trained using TTO and CNN-transfer. We refer to the vectors of importance scores of the kth layer as $v_{TTO}^{\left(k\right)}=\left\{s_{1,TTO}^{\left(k\right)},\ldots ,s_{n^{\left(k\right)},TTO}^{\left(k\right)}\right\}\in R^{n^{\left(k\right)}}$ and $v_{CT}^{\left(k\right)}=\left\{s_{1,CT}^{\left(k\right)},\ldots ,s_{n^{\left(k\right)},CT}^{\left(k\right)}\right\}\in R^{n^{\left(k\right)}}$ for TTO and CNN-transfer, respectively. We then applied a min-max normalisation on each $v_{*}^{\left(k\right)}\in R^{n^{\left(k\right)}}$ (with ∗∈{TTO,CT}) to obtain a normalised vector of scores ${\tilde{v}}_{*}^{\left(k\right)}\in R^{n^{\left(k\right)}}$. Because NISP backpropagates only positive scores, the absolute values of neuron importance scores in one layer increase as the layer is closer to the input of the DNN. This normalisation was performed to allow comparison between scores of layers independently of their depth. For the all layers, we finally computed the Euclidean distance between both ${\tilde{v}}_{TTO}^{\left(k\right)}$ and ${\tilde{v}}_{CT}^{\left(k\right)}$ by

(4) $$D^{\left(k\right)}=\left|\right|{\tilde{v}}_{TTO}^{\left(k\right)}-{\tilde{v}}_{CT}^{\left(k\right)}{\left|\right|}_{2}$$

that we refer to as the *difference of the kth layer*. This allows us to determine which layers were the most similar or dissimilar after the training using TTO and CNN-transfer. Figure 5 shows the layer differences $D^{\left(k\right)}$ arranged in decreasing order.

We can observe that, for some layers, the differences in neuron importance scores between TTO and CNN-transfer are fairly significant. Since each layer encodes specific features, this indicates differences in the features learned using either CNN-transfer or TTO. We analysed the features encoded by the layers with the highest score differences between TTO and CNN-transfer. As shown in Figure 3, each layer belonging to one branch processes data coming from a certain device, i.e., smartphone, smartwatch, or smartglasses. We therefore categorised layers depending on the device (as depicted by the colours in Figure 5). Layers not belonging to any branch (e.g., concatenation or fully-connected layers) were categorised as “other”. We observed that layers with the highest differences encode features computed on data coming from the smartwatch.

Preliminary experiments carried out on the CogAge dataset showed that the smartwatch was the most important device for the classification of behavioural activities, which indicates that it also provides the most relevant features. We could confirm this by checking which channels of the input data were the most important to the classification of behavioural activities. For this, we computed the *Jacobian matrix* of the mDNN trained by TTO or CNN-transfer, following an approach similar to [50]. The mDNN estimates the predictive function $f_{T}:R^{L\times S}\to R^{C_{T}}$, where *L* is the length of a multichannel segment *X* belonging to the target dataset $X_{T}$, *S* is the number of channels of this segment, and $C_{T}$ is the number of classes. The multichannel segment $X={\left(x_{ls}\right)}_{l,s}\in R^{L\times S}$ is a L×S matrix where each element $x_{ls}$ represents the value at the lth time point (1≤l≤L) of the sth sensor channel (1≤s≤S). In addition, $f_{T}$ associates *X* to a vector of softmax probabilities for $C_{T}$ classes, $f_{T}\left(X\right)=\left(f_{T,1}\left(X\right),\cdots ,f_{T,c}\left(X\right),\cdots ,f_{T,C_{T}}\left(X\right)\right)$ ($1\leq c\leq C_{T}$). Under this setting, a *Jacobian value* is defined as

(5) $$J_{c,l,s}\left(X\right)=\frac{\partial }{\partial x_{ls}}f_{T,c}\left(X\right)$$

It gives the information on how much the variation in $x_{ls}$ affects the softmax probability for the cth class. $J_{c,l,s}\left(X\right)$ can be used to determine which $x_{ls}$ in *X* would matter the most for the classification of *X* into the cth class: the smaller $J_{c,l,s}\left(X\right)$ is (in absolute value), the less impact variations in $x_{ls}$ are, and therefore the less important $x_{ls}$ is. In contrast, $x_{ls}$ associated with higher $J_{c,l,s}\left(X\right)$ (in absolute value) are more important.

We apply this reasoning “channel-wise” to *X*. In particular, we compute a *channel-wise Jacobian score for X*$\omega_{s}\left(X\right)$ as the average of absolute $J_{c,l,s}\left(X\right)$ over all the *L* time points and all the $C_{T}$ classes, that is,

(6) $$\omega_{s}\left(X\right)=\frac{1}{C_{T}}\frac{1}{L}\sum_{c=1}^{C_{T}}\sum_{l=1}^{L}\left|J_{c,l,s}\left(X\right)\right|$$

$\omega_{s}\left(X\right)$ indicates the overall importance of the sth channel for the classification of *X*. Finally, we compute a *global channel-wise Jacobian score* $\Omega_{s}$ by averaging $\omega_{s}\left(X\right)$ over all segments in $X_{T}$:

(7) $$\Omega_{s}=\frac{1}{card(X_{T})}\sum_{X\in X_{T}}\omega_{s}\left(X\right)$$

A high value of $\Omega_{s}$ indicates a high importance of the sth sensor channel for the classification problem.

**Figure 6:**
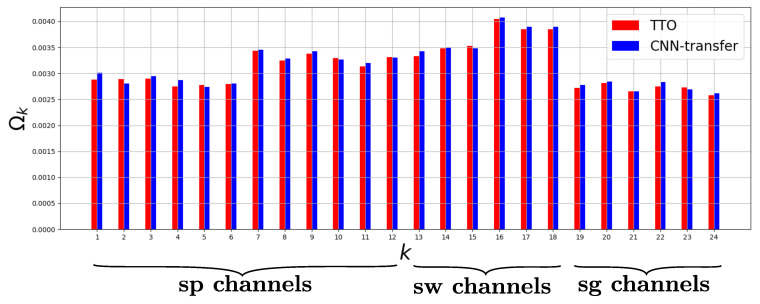
Global channel-wise Jacobian scores $\Omega_{k}$ for mDNNs trained by TTO (red) and CNN-transfer (blue). These scores are computed for BBH on the testing set of the CogAge dataset. *sp*, *sw* and *sg* refer to smartphone, smartwatch, and smartglasses, respectively.

Figure 6 shows the values of $\Omega_{s}$ obtained for both mDNNs trained by TTO and CNN-transfer for the S=24 sensor channels for BBH classification on the CogAge dataset. It can be observed that the scores obtained for both CNN-transfer and TTO do not significantly differ. For both of them, some input sensor channels such as smartphone gyroscope (k∈{7,8,9} in Figure 6) and linear acceleration (k∈{10,11,12}), all smartwatch modalities (k∈{13,14,15,16,17,18}) contribute more to the target task than the others, especially accelerometer and gyroscope of the smartglasses (k∈{19,20,21,22,23,24}). The highest Jacobian scores are obtained for channels of the smartwatch which matches the observations on our preliminary experiments on the CogAge dataset.

The NISP+InfFS and Jacobian experiments for behavioral activity classification on the CogAge dataset showed that the layers of the mDNN processing the most useful sensor channels (Figure 6) also had the largest differences in importance scores between TTO and CNN-transfer (fig:layer-scores). The largest score differences were found in layers processing smartwatch data, which is the device providing the most important data for BBH and BLHO classification for both TTO and CNN-transfer. This indicates that the transferred features on the most important channels were successfully fine-tuned into more discriminative features, while not causing loss of information in the other channels. Our future work will focus on confirming whether this phenomenon also occurs for different target domains by carrying out the same experiments on other target datasets.

## 7. Conclusions

In this paper, we proposed a deep transfer learning approach which could be generally be applied to a variety of classification problem using non-sparse time-series data. It is based on an idea to build a source dataset containing as many different sensor modalities as possible: existing time-series datasets used for various applications are aggregated, segmented, and labelled with their corresponding sensor modality. The source dataset is then used to train a sDNN that encodes general time-series features to perform sensor modality classification. Then, a mDNN is constructed by replicating and fine-tuning the sDNN for each of the sensor channels of the target domain. The architecture of the mDNN allows for handling different target domains regardless of their numbers of sensor channels. Our approach was tested against two baselines—TTO and VAE-transfer—on two very different target domains: wearable-based HAR and ER. For wearable HAR, we also introduced the Cognitive Village-MSBand dataset, a new benchmark dataset for wearable-based HAR.

The results showed that our transfer approach yields the best performances on both tested datasets. This indicates that our method is robust to variations in type and format of the target data. It is also robust to variations in quantity of the training data on the target domain, since our method outperformed the baselines for different amounts of training data on both the CogAge and DEAP datasets. We believe that our method could let researchers bypass the issue of target data scarcity by leveraging existing time-series datasets. Furthermore, our classification experiments on the CogAge and DEAP datasets showed that information relevant to the target problem could in particular be extracted from completely unrelated source datasets. Although further experiments would be needed to confirm whether such results can be reproduced on other target domains, we foresee that our approach could be useful for ubiquitous computing applications, where acquiring large quantities of labelled data for a specific problem is difficult, but a high number of datasets for various applications is available.

Despite the extensive experiments carried out in this paper, the following two points need further investigation: the first one is to expand the scope of our studies by analysing the impact of adding, removing or picking specific sensor modalities and datasets from the source domain. This could give a better assessment of the robustness of our approach. Following an approach similar to [11], we plan to check the influence of the amount of source data, number and granularity of the classes on the source domain. Additionally, adding different types of time-series in the source domain (e.g., sparse time-series, event-based data from *Lifelogging* datasets [51], etc.) could be useful to check whether our approach can also work for target applications not using non-sparse time-series data. Finally, testing its performances on additional target domains could verify its generality in a larger scale, and will be performed in future works.

The second point is to provide a further interpretation of the features transferred from the source to the target domain, and why they allow classification models to perform better than not using transfer on the target domain. Some initial insights have been provided in this paper by computing neuron importance scores (relatively to the target classification) using the NISP and InfFS approaches, and the mDNN Jacobian matrix on the CogAge dataset. Those experiments showed that our transfer approach obtains different and more relevant features than the ones obtained by TTO, by re-adapting the transferred features during the fine-tuning phase. However, our analysis remained on a general level by comparing mDNNs trained by our approach and TTO in a layer-wise fashion. In future work, we will check further how importance scores differ for each layer. In particular, importance scores and their distribution among the neurons of each layer will be analysed to identify which of the features learned on the source domain were the most useful for the target domain.

## Figures and Tables

**Table 1:** List of sensor modalities in the source domain using the OPPORTUNITY, gas-mixture, EEG-Eye-State and energy-appliance datatsets (obtained from the documentation of each respective dataset). The respective units of measurement are provided in parenthesis when the information was available.

Source Dataset	Sensor Modalities	
OPPORTUNITY	· Acceleration (in milli g)	· IMU EU (in degree)
	· IMU magnetometer	· IMU angular velocity (in mm·s${}^{-1}$)
	· IMU gyroscope	· IMU compass (in degree)
	· IMU acceleration (normalised value in milli g)	
gas-mixture	· Gas concentration (in ppm)	· Conductance (in k$\Omega^{-1}$)
EEG-eye-state	· EEG	
energy-appliance	· Energy use (in W·h${}^{-1}$)	· Pressure (in mmHg)
	· Temperature (in ${}^{\circ }$C)	· Wind speed (in m·s${}^{-1}$)
	· Humidity (in %)	· Visibility (in km)

**Table 2:** List of the state and behavioral activities of the Cognitive Village dataset. For activities with a * symbol, executions with either the left or right hand were distinguished.

State activities		
Standing Sitting Lying Squatting Walking Bending		
**Behavioral activities**		
Sit down	Stand up	Lie down
Get up	Squat down	Stand up from squatting
Open door *	Close door *	Open drawer *
Close drawer *	Open small box *	Close small box *
Open big box	Close big box	Open lid by rotation *
Close lid by rotation *	Open other lid *	Close other lid *
Open bag	Take from floor *	Put on floor *
Bring	Put on high position *	Take from high position *
Take out *	Eat small thing *	Drink *
Scoop and put *	Plug in *	Unplug *
Rotate *	Throw out *	Hang
Unhang	Wear jacket	Take off jacket
Read	Write*	Type *
Talk using telephone *	Touch smartphone screen *	Open tap water *
Close tap water *	Put from tap water *	Put from bottle *
Throw out water *	Gargle	Rub hands
Dry off hands by shake	Dry off hands	Press from top *
Press by grasp *	Press switch/button *	Clean surface *
Clean floor		

**Table 3:** Number of 5-second executions for each subset of the CogAge dataset. Executions of session #1 and #2 were respectively used to build the training and testing sets in the subject-dependent setup.

Dataset	Session #1	Session #2
**State**	260	275
**BLHO**	1692	1705
**BBH**	2284	2288

**Table 4:** Number of 5-s executions for each subject of the CogAge dataset.

Dataset	Subject #1	Subject #2	Subject #3	Subject #4
**State**	165	120	120	130
**BLHO**	986	872	718	821
**BBH**	1297	1096	1078	1101

**Table 5:** Accuracies, Average F1-Scores, and MAPs (in %) obtained by TTO, VAE-transfer, and CNN-transfer for the state, BLHO, and BBH classification problems in the subject-dependent configuration. The best performances for each classification problem and evaluation metric are highlighted in bold.

Transfer Approach	State			BLHO			BBH		
	Acc.	AF1	MAP	Acc.	AF1	MAP	Acc.	AF1	MAP
**TTO**	95.91	**95.94**	97.63	71.95	71.72	75.03	67.94	67.65	72.00
**VAE-transfer**	94.78	94.77	**97.93**	64.44	64.09	67.37	61.31	61.04	65.18
**CNN-transfer**	**95.94**	**95.94**	97.62	**76.44**	**76.07**	**79.09**	**71.85**	**71.41**	**75.14**

**Table 6:** Accuracies, Average F1-Scores, and MAPs (in %) obtained by TTO, VAE-transfer, and CNN-transfer for the state, BLHO, and BBH classification problems in the subject-independent configuration (leave-one-subject-out cross-validation). The best average performances for each classification problem and evaluation metric are highlighted in bold.

Transfer Approach	Fold Index	State			BLHO			BBH		
		Acc.	AF1	MAP	Acc.	AF1	MAP	Acc.	AF1	MAP
**TTO**	**1**	87.27	86.95	93.73	33.81	32.23	31.87	29.77	26.52	29.96
	**2**	91.67	91.57	95.19	52.47	50.37	52.32	46.45	44.01	46.24
	**3**	95.99	94.16	95.99	34.61	30.51	33.55	30.58	28.74	32.93
	**4**	90.66	90.82	97.15	55.52	52.01	56.96	47.69	44.27	47.71
	**Average**	**91.40**	**90.88**	**95.52**	44.10	41.28	43.68	38.62	35.89	39.21
	**Standard-deviation**	3.59	2.98	1.44	11.50	11.48	12.82	9.77	9.58	9.07
**VAE-transfer**	**1**	83.58	83.13	88.13	35.02	31.62	30.35	31.74	28.61	28.02
	**2**	90.00	89.66	93.26	51.58	47.99	52.65	43.48	42.09	43.89
	**3**	95.00	94.95	98.59	35.18	32.06	36.84	29.01	28.54	31.51
	**4**	82.58	76.35	93.79	54.43	50.03	52.54	48.23	43.61	46.38
	**Average**	87.79	86.02	93.45	44.05	40.43	43.10	38.12	35.71	37.45
	**Standard-deviation**	5.82	8.06	4.28	10.40	9.95	11.29	9.21	8.27	9.04
**CNN-transfer**	**1**	88.21	87.56	92.40	36.33	33.99	35.39	33.57	30.05	31.07
	**2**	84.17	83.89	92.58	52.98	49.34	53.92	47.23	45.33	51.15
	**3**	98.33	98.33	98.01	38.28	33.66	40.66	32.94	32.52	36.03
	**4**	91.52	91.56	96.60	58.85	55.40	62.04	50.19	46.60	53.86
	**Average**	90.56	90.34	94.90	**49.39**	**43.10**	**48.00**	**40.98**	**38.63**	**43.03**
	**Standard-deviation**	5.99	6.18	2.84	11.68	10.99	12.18	9.01	8.55	11.18

**Table 7:** 10-fold cross validation accuracies (in %) for the classification of AROUSAL using a multichannel DNN on the DEAP dataset. $F_{i}$ refers to fold number *i*. The best performance for each fold is highlighted in bold.

Approach	F1	F2	F3	F4	F5	F6	F7	F8	F9	F10	Average
**Bi-modal AE [7]**	-	-	-	-	-	-	-	-	-	-	80.50
**TTO**	89.26	88.13	87.55	87.69	88.13	88.05	88.64	88.75	87.59	87.91	88.17
**VAE-transfer**	83.22	84.87	84.93	85.04	83.74	84.86	84.71	84.55	85.12	83.93	84.50
**CNN-transfer**	**90.89**	**91.60**	**91.18**	**91.46**	**91.37**	**91.53**	**90.79**	**91.59**	**91.64**	**90.80**	**91.29**

**Table 8:** 10-fold cross validation accuracies (in %) for the classification of VALENCE using a multichannel DNN on the DEAP dataset. $F_{i}$ refers to fold number *i*. The best performance for each fold is highlighted in bold.

Approach	F1	F2	F3	F4	F5	F6	F7	F8	F9	F10	Average
**Bi-modal AE [7]**	-	-	-	-	-	-	-	-	-	-	85.20
**TTO**	87.67	87.03	87.85	86.93	87.26	87.44	88.03	87.08	87.75	87.25	87.43
**VAE-transfer**	85.17	84.86	83.92	85.48	84.75	84.06	84.23	85.42	84.90	84.69	84.75
**CNN-transfer**	**90.89**	**91.12**	**90.22**	**90.39**	**90.51**	**90.27**	**90.71**	**90.39**	**91.08**	**90.84**	**90.64**

**Table 9:** Accuracies, AF1s, and MAPs (in %) of TTO, VAE-transfer, and CNN-transfer after downsampling of the training set, for the classification of state, BLHO, and BBH activities on the CogAge dataset (subject-dependent configuration).

Transfer Approach	Training Target Data Proportion (%)	State			BLHO			BBH		
		Acc.	AF1	MAP	Acc.	AF1	MAP	Acc.	AF1	MAP
**TTO**	**5**	66.56	61.51	68.70	19.05	16.24	16.62	19.86	15.31	15.82
	**25**	88.78	88.92	94.16	51.98	51.23	50.90	50.83	50.21	52.67
	**50**	93.78	93.67	96.88	60.89	60.74	62.07	58.90	58.39	61.99
	**75**	95.14	95.18	97.73	71.11	71.24	73.18	62.78	62.70	65.99
	**100**	95.91	95.94	97.63	71.95	71.72	75.03	67.94	67.65	72.00
**VAE** **transfer**	**5**	59.79	57.44	64.55	19.05	14.88	17.43	15.11	13.03	13.49
	**25**	89.14	88.92	91.42	39.20	37.34	38.53	32.92	32.46	33.00
	**50**	89.09	89.05	95.69	51.91	51.52	52.79	47.52	47.36	49.18
	**75**	94.84	94.81	97.91	61.45	60.58	62.35	51.29	50.60	53.90
	**100**	94.78	94.77	97.93	64.44	64.09	67.37	61.31	61.04	65.18
**CNN** **transfer**	**5**	67.64	67.70	73.74	23.39	18.10	20.41	25.92	22.05	24.13
	**25**	90.47	90.61	94.12	55.89	55.46	56.97	56.95	55.65	57.51
	**50**	94.60	94.47	97.73	66.66	66.01	68.59	62.83	62.22	66.22
	**75**	95.88	95.87	97.11	73.06	72.47	74.99	66.29	66.29	69.95
	**100**	95.94	95.94	97.62	76.44	76.07	79.09	71.85	71.41	75.14

**Table 10:** 10-fold cross validation accuracies (in %) of TTO, VAE-transfer, and CNN-transfer after downsampling of the training set for the classification of AROUSAL on the DEAP dataset. $F_{i}$ refers to fold number *i*.

Transfer Approach	Training Target Data Proportion (%)	F1	F2	F3	F4	F5	F6	F7	F8	F9	F10	Average
**TTO**	**5**	64.11	64.81	64.00	63.64	63.81	65.49	65.74	67.44	64.62	64.61	64.83
	**25**	78.47	76.28	76.08	76.66	76.75	77.53	74.91	76.92	77.80	76.23	76.76
	**50**	82.08	81.56	82.29	84.34	82.03	83.94	82.68	82.42	82.90	83.16	82.74
	**75**	85.88	87.49	87.25	85.16	87.05	85.13	85.59	86.22	86.86	85.88	86.25
	**100**	89.26	88.13	87.55	87.69	88.13	88.05	88.64	88.75	87.59	87.91	88.17
**VAE** **transfer**	**5**	63.52	64.26	64.20	65.80	64.83	64.24	64.42	64.91	64.04	63.70	63.70
	**25**	74.12	74.79	74.59	74.78	74.45	74.63	73.76	74.49	74.09	73.67	74.34
	**50**	78.99	79.92	80.31	80.05	79.50	80.25	79.49	79.34	79.77	79.12	79.67
	**75**	82.14	83.07	82.56	83.12	82.54	82.13	82.48	82.69	82.19	82.25	82.52
	**100**	83.22	84.87	84.93	85.04	83.74	84.86	84.71	84.55	85.12	83.93	84.50
**CNN** **transfer**	**5**	65.66	65.58	65.91	66.64	65.71	65.45	66.94	65.31	66.90	66.48	66.06
	**25**	79.86	80.47	79.97	80.34	79.59	78.91	79.58	80.05	80.39	80.18	79.93
	**50**	86.79	86.95	86.32	86.77	86.48	87.54	86.41	86.58	86.78	86.76	86.74
	**75**	89.37	89.66	89.46	90.60	89.72	89.74	89.12	89.55	89.66	88.81	89.57
	**100**	90.89	91.60	91.18	91.46	91.37	91.53	90.79	91.59	91.64	90.80	91.29

**Table 11:** 10-fold cross validation accuracies (in %) of TTO, VAE-transfer, and CNN-transfer after downsampling of the training set for the classification of VALENCE on the DEAP dataset. $F_{i}$ refers to fold number *i*.

Transfer Approach	Training Target Data Proportion (%)	F1	F2	F3	F4	F5	F6	F7	F8	F9	F10	Average
**TTO**	**5**	64.27	64.36	64.29	62.71	63.86	63.50	63.08	65.17	63.76	62.79	63.78
	**25**	77.78	75.60	76.33	76.23	76.26	74.88	75.93	76.12	76.44	76.07	76.16
	**50**	82.49	81.78	81.89	81.88	81.63	82.61	81.36	81.87	82.76	83.21	82.15
	**75**	85.42	85.97	84.76	85.64	85.21	85.62	84.92	86.21	85.13	86.04	85.49
	**100**	87.67	87.03	87.85	86.93	87.26	87.44	88.03	87.08	87.75	87.25	87.43
**VAE** **transfer**	**5**	64.11	64.43	63.29	65.05	64.55	65.22	64.14	65.35	64.72	65.38	64.62
	**25**	74.67	73.57	74.31	74.70	73.26	74.32	74.52	74.02	74.48	74.18	74.20
	**50**	80.41	80.11	79.94	80.49	80.13	80.11	80.12	80.53	81.05	79.33	80.22
	**75**	83.79	82.68	82.73	83.95	83.33	82.68	82.78	82.85	83.41	82.96	83.12
	**100**	85.17	84.86	83.92	85.48	84.75	84.06	84.23	85.42	84.90	82.96	83.12
**CNN** **transfer**	**5**	65.31	64.94	65.48	64.16	63.96	64.19	65.45	65.25	65.43	65.15	64.93
	**25**	80.27	79.91	78.81	79.01	79.50	80.01	79.32	81.47	79.45	80.38	79.81
	**50**	86.44	86.37	85.60	86.77	85.16	85.71	85.54	85.68	86.74	86.71	86.07
	**75**	88.86	89.43	89.09	88.98	89.35	88.75	89.01	89.53	88.85	89.45	89.13
	**100**	90.89	91.12	90.22	90.39	90.51	90.27	90.71	90.39	91.08	90.84	90.64

## Supplementary Materials

The datasets and codes used for the studies presented in this paper are available online at https://www.info.kindai.ac.jp/~shirahama/transfer/.
